# Ultrafast Spectroscopy and Red Emission from β-Ga_2_O_3_/β-Ga_2_S_3_ Nanowires

**DOI:** 10.1186/s11671-015-1016-y

**Published:** 2015-07-28

**Authors:** Katerina M Othonos, Matthew Zervos, Constantinos Christofides, Andreas Othonos

**Affiliations:** Laboratory of Ultrafast Science, Department of Physics, University of Cyprus, PO Box 20537, Nicosia, 1678 Cyprus; Nanostructured Materials and Devices Laboratory, Department of Mechanical and Manufacturing Engineering, University of Cyprus, P.O. Box 20537, Nicosia, 1678 Cyprus; Neuroscience, University of British Columbia, Vancouver, Canada

**Keywords:** β-Ga_2_O_3_/Ga_2_S_3_ nanowires, Pump-probe spectroscopy, Carrier dynamics, Photoluminescence

## Abstract

Ultrafast pump-probe and transient photoluminescence spectroscopy were used to investigate carrier dynamics in β-Ga_2_O_3_ nanowires converted to β-Ga_2_O_3_/Ga_2_S_3_ under H_2_S between 400 to 600 °C. The β-Ga_2_O_3_ nanowires exhibited broad blue emission with a lifetime of 2.4 ns which was strongly suppressed after processing at 500–600 °C giving rise to red emission centered at 680 nm with a lifetime of 19 μs. Differential absorption spectroscopy reveals that state filling occurs in states located below the conduction band edge before sulfurization, but free carrier absorption is dominant in the β-Ga_2_O_3_/Ga_2_S_3_ nanowires processed at 500 to 600 °C for probing wavelengths >500 nm related to secondary excitation of the photo-generated carriers from the mid-gap states into the conduction band of Ga_2_S_3_.

## Background

Metal oxide (MO) semiconductor nanowires (NWs) such as SnO_2_, In_2_O_3_, and Sn-doped In_2_O_3_, have been investigated extensively for the fabrication of nanoscale devices such as sensors and nanowire solar cells [1, 2]. With the incorporation of MO NWs in such devices that are subjected to various gasses such as NH_3_, H_2_S, or liquids containing S, Na_2_S requires an understanding of their effect on the electrical, optical, and structural properties of the MO NWs. In the past, we investigated the growth and properties of SnO_2_ [3], In_2_O_3_ [4], Sn-doped In_2_O_3_ [5], and β-Ga_2_O_3_ NWs and found that they exhibit photoluminescence at ≈ 2.5 eV due to oxygen vacancies and states residing energetically in the upper half of the energy band gap as shown by ultrafast absorption spectroscopy [6].

More recently, we showed that post growth processing of SnO_2_ and Sn-doped In_2_O_3_ NWs under H_2_S between 300 to 600 °C resulted in an increase of conductivity and band edge emission, respectively. However, there are no investigations into the conversion of β-Ga_2_O_3_ into β-Ga_2_O_3_/Ga_2_S_3_ NWs under H_2_S. Ga_2_S_3_ is a III-VI semiconductor which can have the monoclinic, hexagonal, or cubic crystal structure [7]. Among these, the most stable crystal structure is monoclinic of Ga_2_S_3_ which has a direct wide-band gap of 3.4 eV [8, 9] but contains many vacancies in its Ga sub lattice and consequently exhibits defect emission similar to oxides [10, 11]. Despite this, Ga_2_S_3_ has been investigated for light-emitting diodes and photovoltaic devices while recently, it was shown that Ga_2_S_3_ has a large second-harmonic generation efficiency ideally suited for nonlinear optics [12]. The optical properties of single crystals and epitaxial layers of Ga_2_S_3_ have been investigated using steady-state absorption-transmission and photoluminescence (PL) spectroscopy and exhibit blue and red emission at 10 K due to deep donor-to-acceptor transitions related to S and Ga vacancies [8, 10, 11]. In contrast, one-dimensional GaS or Ga_2_S_3_ nanotubes so far have been shown to exhibit PL between 450–600 nm [13–15], but there are no detailed investigations on the optical properties of GaS or Ga_2_S_3_ NWs using steady-state or transient optical spectroscopy which could provide insight into their fundamental properties and potential device applications.

Consequently, we have undertaken an investigation into the post growth processing of β-Ga_2_O_3_ NWs under H_2_S between 400–600 °C and its effect on the optical properties using ultrafast absorption spectroscopy in conjunction with steady-state and time-resolved PL. We find that the as-grown β-Ga_2_O_3_ NWs exhibit state filling following carrier excitation at 266 nm (*λ*_E_) for probing wavelengths (*λ*_p_) between 340 to 450 nm corresponding to states lying energetically below but near the conduction band edge. In addition, the β-Ga_2_O_3_ NWs exhibited broad PL with a maximum at 435 nm and lifetime of 2.4 ns. Similar results have also been observed for the β-Ga_2_O_3_/Ga_2_S_3_ NWs after post growth processing under H_2_S at 400 °C. In contrast, the β-Ga_2_O_3_/Ga_2_S_3_ NWs obtained under H_2_S at 500 to 600 °C exhibit free carrier absorption for *λ*_p_ > 500 nm and state filling for *λ*_p_ < 450 nm related to the formation of Ga_2_S_3_. This is accompanied by a suppression of the broad PL at 435 nm and the emergence of strong emission at ≈ 680 nm with a much longer lifetime of 19 μs. In addition, at low temperature, a relative narrow emission at 428 nm emerges resulting from near band edge states to the acceptor states located near the valance band of the β-Ga_2_S_3_.

## Methods

β-Ga_2_O_3_ NWs were grown by low-pressure chemical vapor deposition at 800 °C and 1mBar for 60 min on Si(001) using a 1-nm layer of Au as catalyst and exactly the same growth conditions used for Sn-doped In_2_O_3_ NWs as described in detail elsewhere [5]. The morphology of the NWs were then examined with a TESCAN scanning electron microscope (SEM) while their crystal structure and phase purity were investigated using a SHIMADZU, XRD-6000, X-ray diffractometer with Cu-Ka source, by performing a scan of θ-2θ in the range between 10° and 80°. The dynamic behavior of carriers within the NWs was investigated through the temporal behavior of ultrafast time-resolved differential absorption obtained from simultaneous measurements of time-resolved differential transmission and reflection [16, 17]. The experiments were carried out using a Ti: Sapphire ultrafast amplifier system generating 100-fs pulses at 800 nm and running at a repetition rate of 1 kHz. Nonlinear crystals were used to generate 266 nm for the purpose of exciting the NWs whereas part of the fundamental and second-harmonic generation at 400 nm was used to generate a super continuum light for probing different energy states. Measurements were carried out using a typical pump-probe optical setup in a non collinear configuration. Photoluminescence were carried out utilizing a spectrometer equipped with an intensified charge-coupled device camera. The low-temperature measurements were carried out using a closed cycle refrigerator capable of cooling the sample to temperatures below 10 K.

## Results and Discussion

**Fig. 1:**
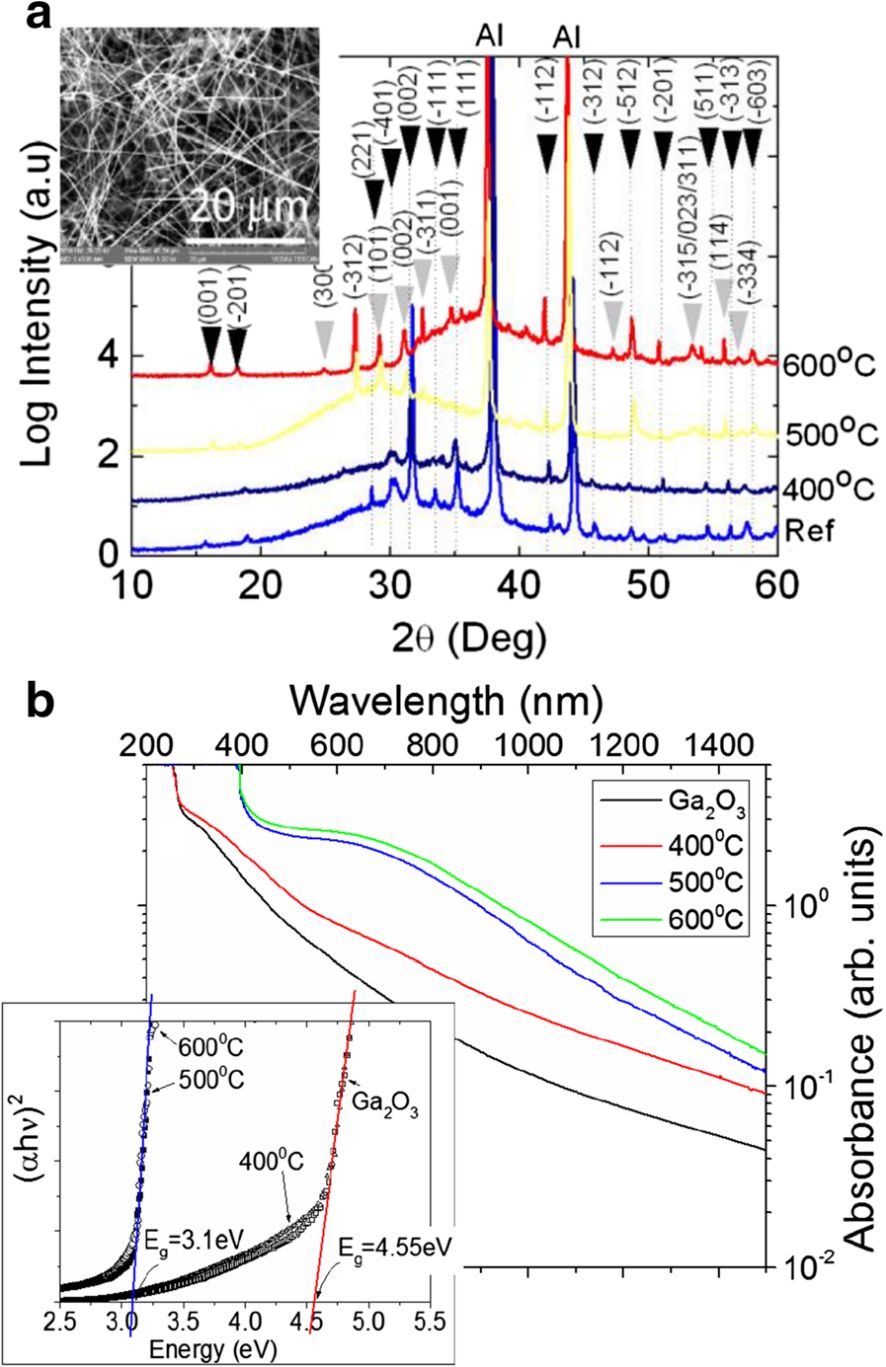
**a** XRD of β-Ga_2_O_3_ NWs before and after post growth processing under H_2_S at 400, 500, and 600 °C for 60 min. *Black arrows* at the top correspond to β-Ga_2_O_3_ and *gray* to β-Ga_2_S_3_ while the Al peaks belong to the holder; *inset* shows a SEM image of the β-Ga_2_S_3_ /β-Ga_2_O_3_ NWs at 500 °C. **b** Steady-state transmission of as-grown β-Ga_2_O_3_ NWs at 300 K and β-Ga_2_O_3_/Ga_2_S_3_ NWs obtained under H_2_S at 400, 500, and 600 °C. The optical band gap is estimated from the *x* intercept of the *inset*, where (*α*hν)^2^ is plotted as a function of photon energy

A typical SEM image of the β-Ga_2_O_3_ NWs which had diameters of 50–100 nm and lengths of up to 100 μm is shown as an inset in Fig. 1a.

The β-Ga_2_O_3_ NWs have a monoclinic crystal structure similar to that obtained previously [6] and were exposed to 50 sccm of H_2_S at 400, 500, and 600 °C for 60 min using a ramp rate of 10 °C/min which resulted in the formation of the β-Ga_2_O_3_/β-Ga_2_S_3_ NWs as shown by the XRD of Fig. 1a. In particular, we observe peaks belonging to β-Ga_2_O_3_ but also the emergence of monoclinic β-Ga_2_S_3_ due to the diffusion of S into β-Ga_2_O_3_. Ga_2_S_3_ is a III-VI defect semiconductor which can have the monoclinic, hexagonal, or cubic crystal structure. Among these, the most stable crystal structure of Ga_2_S_3_ is the monoclinic phase. We observe a suppression of most peaks belonging to β-Ga_2_O_3_ with increasing temperature although a few remain at 600 °C which means that high temperatures are necessary to achieve complete conversion into Ga_2_S_3_. Interestingly, the β-Ga_2_O_3_/β-Ga_2_S_3_ NWs remained one-dimensional up to 600 °C in contrast to SnO_2_ and Sn-doped In_2_O_3_ NWs which were eliminated under H_2_S due to their reduction by H_2_ [18, 19]. Here, we should point out that post growth processing of MO NWs such as Sn-doped In_2_O_3_ at elevated temperatures resulted n the formation of crystals on the surface and not a conformal or epitaxial like shell [19]. The lattice constants of β-Ga_2_O_3_ are *a* = 12.23 Å, *b* = 3.04 Å, and *c* = 5.80 Å with β = 103.7° while β-Ga_2_S_3_ have lattice constants of *a* = 11.11 Å, *b* = 9.58 Å, and *c* = 6.4 Å with β **=** 141.15°, so one expects interfacial stress due to the lattice mismatch between the β-Ga_2_O_3_ and β-Ga_2_S_3_. The band line-up and the effect of strain on the β-Ga_2_O_3_/Ga_2_S_3_ heterojunction is not known, but the energy band gap and work function of Ga_2_O_3_ is 4.5 eV and 4.1 eV, respectively [20], while the energy band gap of Ga_2_S_3_ is smaller and equal to 3.42 eV. Assuming that the work functions are similar, the β-Ga_2_O_3_/Ga_2_S_3_ heterojunction is expected to be type I. It is also useful to note that F.Säuberlich et al. [21] showed that the large interface dipole moments exist between metal oxides and chalcogenides resulting into small conduction band discontinuities. Steady-state absorption measurements (Fig 1b) through the NWs grown on fused silica suggest a band gap at ≈ 4.55 eV for the β-Ga_2_O_3_ and 400 °C NWs which decreased to 3.1 eV after post growth processing under H_2_S at 500 and 600 °C consistent with the formation of β-Ga_2_S_3_. Furthermore, there appears to be a broad absorption below the band edge for all the samples most likely due to the defect states present. Unfortunately, the absorption spectra for the β-Ga_2_O_3_/β-Ga_2_S_3_ NWs do not provide any clear evidence of the type of band offset that may exist between this hetero-structure [22]. Nevertheless, transient *PL* and *differential absorption* measurements indicate that the main contribution of both signals is coming from Ga_2_S_3_ suggesting a type I heterojunction. This is confirmed since the observed time constants in the decays in these signals remain the same with increasing post growth processing temperature from 500 to 600 °C and higher where the NW structure is completely converted to Ga_2_S_3_.

**Fig. 2:**
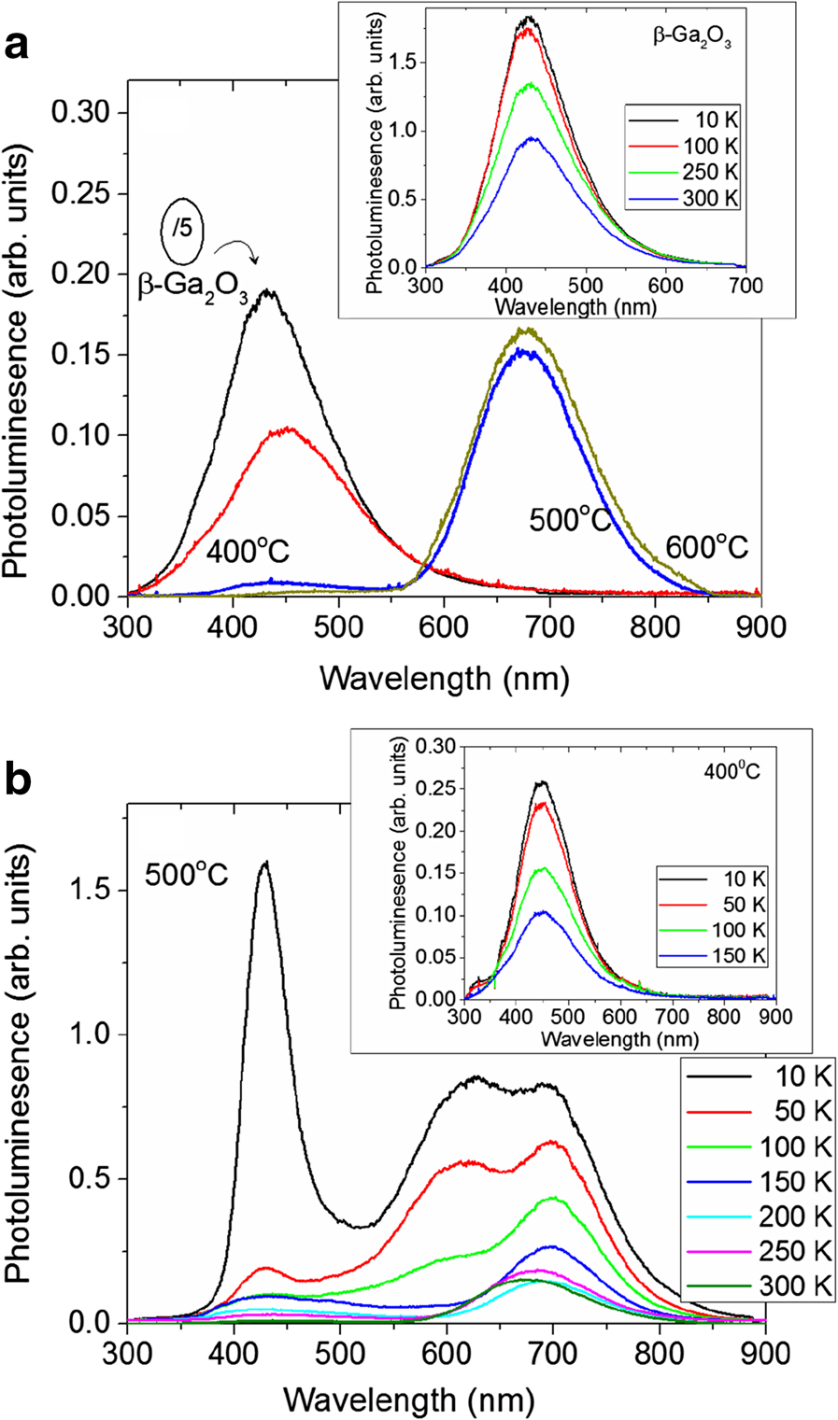
**a** PL of as-grown β-Ga_2_O_3_ NWs at 300 K and β-Ga_2_O_3_/Ga_2_S_3_ NWs obtained under H_2_S at 400, 500, and 600 °C using *λ*_E_ *=* 266 nm; *inset* shows the temperature-dependent PL of the as-grown β-Ga_2_O_3_ NWs, whereas **b** and its *inset* shows temperature-dependent PL of the β-Ga_2_O_3_/Ga_2_S_3_ NWs at 500 and 400 °C, respectively

Photoluminescence of the as-grown β-Ga_2_O_3_ and β-Ga_2_O_3_/Ga_2_S_3_ NWs obtained at 400, 500, and 600 °C is shown in Fig 2a. The as-grown β-Ga_2_O_3_ NWs exhibit broad PL with a maximum at ≈ 435 nm, which is attributed to transitions between donor-like states related to oxygen vacancies (V_O_) and acceptor-like states due to gallium vacancies (V_Ga_) or gallium-oxygen vacancy pairs (V_Ga_-V_O_) [23]. We observe a small red shift of the PL from 2.9 to 2.8 eV after post growth processing under H_2_S at 400 °C and a tenfold reduction in intensity. However, the suppression of the PL at ≈ 2.9 eV was accompanied by the emergence of red emission at ≈ 1.8 eV following post growth processing of the β-Ga_2_O_3_ NWs under H_2_S at 500 and 600 °C as shown in Fig. 2a.

This red emission is attributed to transitions between donor-like states, located ≈ 1.1 eV below the conduction band edge, to acceptor states ≈ 0.4 eV above the valance band edge in Ga_2_S_3_ [10, 11]. It should be noted that the PL of the as-grown β-Ga_2_O_3_ NWs increased by a factor of two upon decreasing the temperature down to 10 K (inset Fig 2a). On the other hand, we observed a sixfold increase of the 680-nm peak for the NWs processed under H_2_S at 500 and 600 °C, whereas new peaks appear at 428 nm (2.9 eV) and 620 nm (2.0 eV) for temperatures below 100 K as shown in Fig. 2b.

The temperature dependence of the PL peak intensity can be described by [24]

$$ I(T)=I(0)/\left[1+C \exp \left(\frac{-{E}_{act}}{k_BT}\right)\right] $$

where *I*(*T*) is the intensity as a function of temperature *T*, *C* is a constant, k_B_ is Boltzmann constant, and *E*_act_ is the activation energy. A fit of this equation to the data provided an activation energy of 18 meV for the 428-nm PL peak.

Given the activation energy, we believe that the emission occurs from states just below the band edge to acceptor states located around the 0.4 eV band. Our findings are consistent with those described by Yoon et al. [10] for Ga_2_S_3_ who identified at 10 K two transitions of 1.93 and 2.9 eV between an acceptor state at 0.42 eV above the valence band edge and donor states at 0.1 eV and 1.1 eV below the conduction band. Furthermore, it appears from our findings that the occupation of the lower-end energy states in the 0.4 eV band at low temperatures results in a substantial increase in the 680-nm line as well as the new emission emerging at 620 nm at <100 K. These trends are consistent with the findings of Ho and Chen [8] who observed PL at 1.99 and 2.79 eV at low temperatures attributed to the formation of sulfur vacancies (V_S_) as well as the existence of Ga vacancies (V_Ga_) which form donor and acceptor levels, respectively. We should point out that in their work on the high quality Ga_2_S_3_ crystal [8], they have observed a broad emission around 500 nm at room temperature in contrast to the observed emission at 680 nm from the β-Ga_2_O_3_/Ga_2_S_3_ NWs (Fig. 2). It should be emphasized that all previous investigations on one-dimensional GaS and α-Ga_2_S_3_ showed PL between 450–600 nm or 2.1–2.8 eV, so here, we show that β-Ga_2_O_3_/Ga_2_S_3_ can exhibit red emission at room temperature which makes them attractive not only for the applications in sensors and non-toxic biomedical imaging but also for down-conversion in solar cells.

**Fig. 3:**
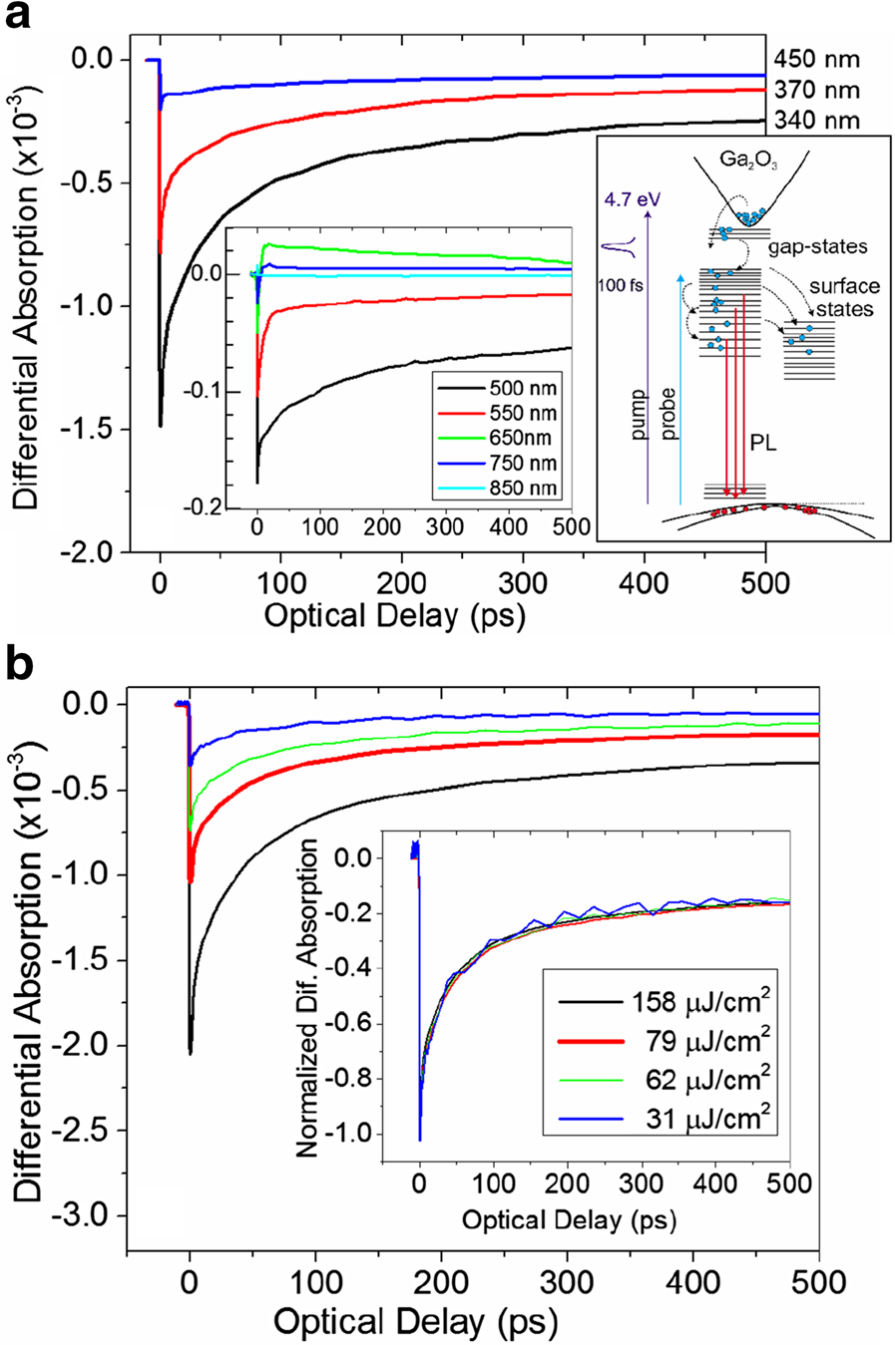
**a** Differential absorption of the as-grown β-Ga_2_O_3_ NWs versus optical delay using *λ*_E_ *=* 266 nm and *λ*_p_ *=* 340 to 850 nm. The diagram shows the various processes following the photo-excitation of the β-Ga_2_O_3_ NWs; *inset* shows traces for *λ*_p_ *>*500 nm on an expanded scale (the same units). **b** Differential absorption measurements with *λ*_p_ *=* 340 nm in β-Ga_2_O_3_ NWs for a range of fluences. The *inset* shows the normalized differential absorption for comparison

**Fig. 4:**
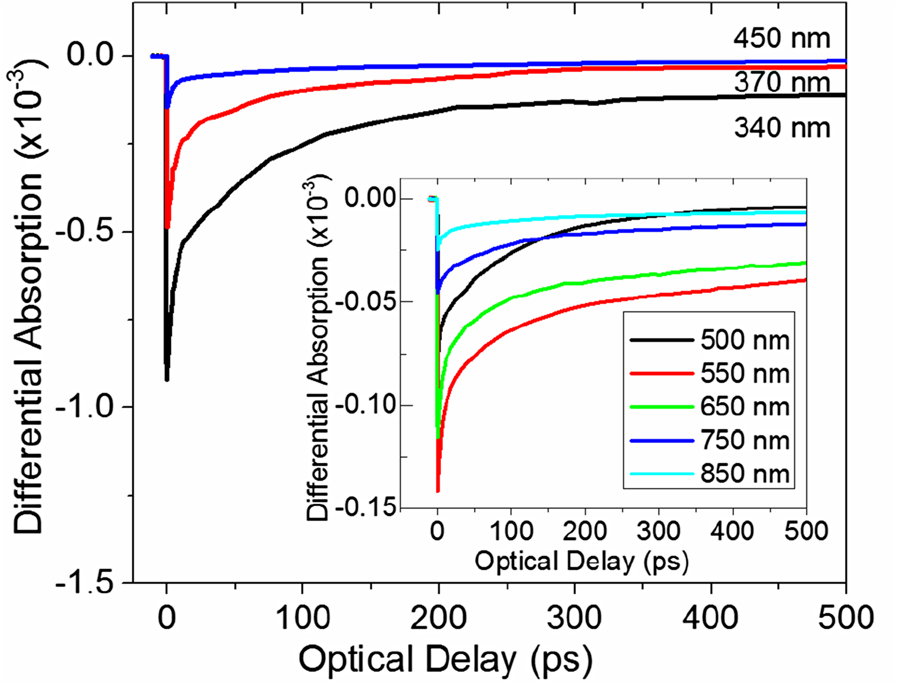
Differential absorption through β-Ga_2_O_3_ NWs after processing under H_2_S at 400 °C versus optical delay using *λ*_E_ = 266 nm and *λ*_p_ *=* 340 to 850 nm; *inset* shows traces for *λ*_p_ *>* 500 nm on an expanded scale

**Fig. 5:**
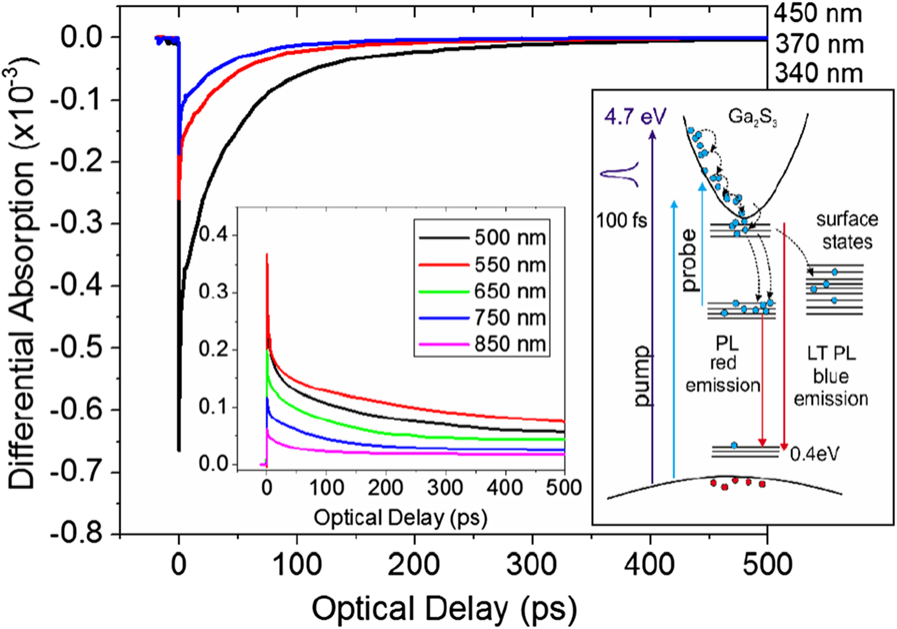
Differential absorption through β-Ga_2_O_3_ NWs grown on fused silica after processing with H_2_S at 500 °C versus optical delay using *λ*_E_ *=* 266 nm and *λ*_p_ *=* 340 to 850 nm. The *inset* shows a schematic energy band diagram and the various processes following photo-excitation of the β-Ga_2_O_3_/Ga_2_S_3_ NWs

In order to gain a better understanding of the optical properties, time-resolved differential absorption measurements were carried out using excitation at 266 nm and probing wavelengths between 340 to 850 nm. The differential absorption versus optical delay acquired from the as-grown β-Ga_2_O_3_ NWs and the Ga_2_O_3_/Ga_2_S_3_ NWs obtained after processing with H_2_S at 400 and 500 °C are shown in Figs. 3, 4, and 5, respectively.

The various traces in each case correspond to different *λ*_p_ over a time span of 10 to 500 ps and were obtained using a fluence of 115 μJ/cm^2^. The value of the fluence was chosen for best signal-to-noise ratio with negligible nonlinear recombination as clearly seen from the normalized fluence measurements in the inset of Fig 3b. Similar behavior was observed for the different probing wavelengths and samples for this range of fluence.

For the shortest probing wavelengths, we observe a pulse width-limited, sharp decrease in the induced absorption followed by a recovery toward equilibrium over a time scale of hundreds of ps. These changes in absorption are associated with the excitation of electrons and holes in the β-Ga_2_O_3_ and Ga_2_O_3_/Ga_2_S_3_ NWs by photons resulting in the generation of non-equilibrium carriers. These non-equilibrium carriers will re-distribute themselves among energy states that are normally unoccupied under equilibrium conditions. This will appear as a decrease in the absorption (state filling) at the energy states that are being probed. The recovery of this absorption change will be a direct measure of the time required by the photo-generated carriers to move out of the occupied states. In the case of the as-grown β-Ga_2_O_3_ NWs, we observe a strong negative differential absorption for *λ*_p_ < 450 nm corresponding to state filling of the energy states located below the conduction band of the β-Ga_2_O_3_ NWs as shown in Fig. 3. The relaxation of the non-equilibrium carriers can be described by a tri-exponential decay with time constants (*τ*) and amplitudes (*α*) of *τ*_1_ = 2–4 ps (*α*_1_ = 28–35 %), *τ*_2_ = 68–85 ps (*α*_2_ = 31–50 %) and *τ*_3_ = 1915–2050 ps (*α*_3_ = 20–41 %). Both *τ*_1_ and *τ*_2_ are related to non-radiative, re-distribution of photo-generated carriers in point defect or surface states, depicted schematically in the energy band diagram shown as an inset in Fig. 3, whereas *τ*_3_ is most likely associated with radiative recombination. For *λ*_p_ = 550 to 600 nm, the observed differential absorption is relatively small and is associated with the Au plasmon resonance [6]. For longer *λ*_p_, the signal appears to be weaker and is associated with what is known as “free carrier absorption”. We should point out here that free carrier absorption is considered as a competing process to state filling, and it occurs due to secondary excitation of the photo-generated carriers by the probing photons from their initial states to higher energy states. This phenomenon will result in a positive change in the induced absorption.

Given that state filling is observed for *λ*_p_ corresponding to energies that are smaller than the energy band gap of β-Ga_2_O_3_, it is evident that the β-Ga_2_O_3_ NWs contain states residing energetically in the upper half of the gap consistent with the broad PL shown in Fig. 2.

Similar trends were also observed in Fig. 4 for the β-Ga_2_O_3_/Ga_2_S_3_ NWs obtained after processing under H_2_S at 400 °C which resulted into a slight reduction of differential absorption and a faster decay of electrons occupying energetically higher states, compared to the as-grown β-Ga_2_O_3_ NWs.

The decay times are similar, i.e., *τ*_1_ = 3.5–5.5 ps (*α*_1_ = 50–52 %), *τ*_2_ = 59–95 ps (*α*_2_ = 34–41 %) and *τ*_3_ = 1950–2010 ps (*α*_3_ = 10–22 %), but the strength of *τ*_1_ increased from 30 to 50 % consistent with the suppression of radiative recombination and blue PL which occurs upon processing the β-Ga_2_O_3_ NWs at 400 °C as shown in Fig. 2. This is also consistent with the fact that there is noticeable state filling observed for longer *λ*_p_ in contrast to the as-grown β-Ga_2_O_3_ NWs, and it is clearly due to the formation of defect states.

Differential absorption measurements for the β-Ga_2_O_3_/Ga_2_S_3_ NWs after exposure to H_2_S at 500 °C are shown in Fig. 5. Again, state filling is observed for *λ*_p_ < 450 nm, but in this case, the main signal is coming from the β-Ga_2_S_3_ where the carriers are generated above the conduction band and relaxation takes place on a very short time scale with *τ*_1_ = 1 ps (*α*_1_ = 25–35 %), *τ*_2_ = 35 ps (*α*_2_ = 34–50 %) and *τ*_3_ = 145–167 ps (*α*_3_ = 8–20 %).

For the larger probing wavelengths, (*λ*_p_) free carrier absorption is dominant. It appears that the β-Ga_2_S_3_ formed during the processing of the as-grown β-Ga_2_O_3_ NWs under H_2_S plays a crucial role in the carrier dynamics. The differential absorption signal for *λ*_p_ > 500 nm appears to be much larger than the contribution from the β-Ga_2_O_3_. The donor states in β-Ga_2_S_3_ [10, 11] which are located approximately 1.1 eV from the conduction band edge are populated following excitation, and as a result, the carriers undergo a secondary excitation by the probing beam back into the conduction band. Further evidence of this was observed for the β-Ga_2_O_3_/Ga_2_S_3_ NWs obtained at 600 °C where differential absorption measurements reveal an increase in the free carrier absorption signal in the long wavelength range. The free carrier absorption may be described well with a two exponential function (*α*_0_ + *α*_1_*e*^-*t*/*τ*^^1^ + α_2_*e*^-*t*/*τ*^^2^) and a constant *α*_0_ associated with the very long radiative emission, consistent with the lifetime of the observed red emission (~19 μs) from the 1.1-eV donor state. The time constants have a range of values of *τ*_1_ = 1.2–2.9 ps (*α*_1_ = 22–25 %), *τ*_2_ = 49–87 ps (*α*_2_ = 49–54 %) and a non-zero *α*_0_ constant (21–29 %). Here, we should also point out that the low-temperature pump-probe measurements carried out in these samples revealed similar temporal behavior with time constants approximately 10-15 % longer.

**Fig. 6:**
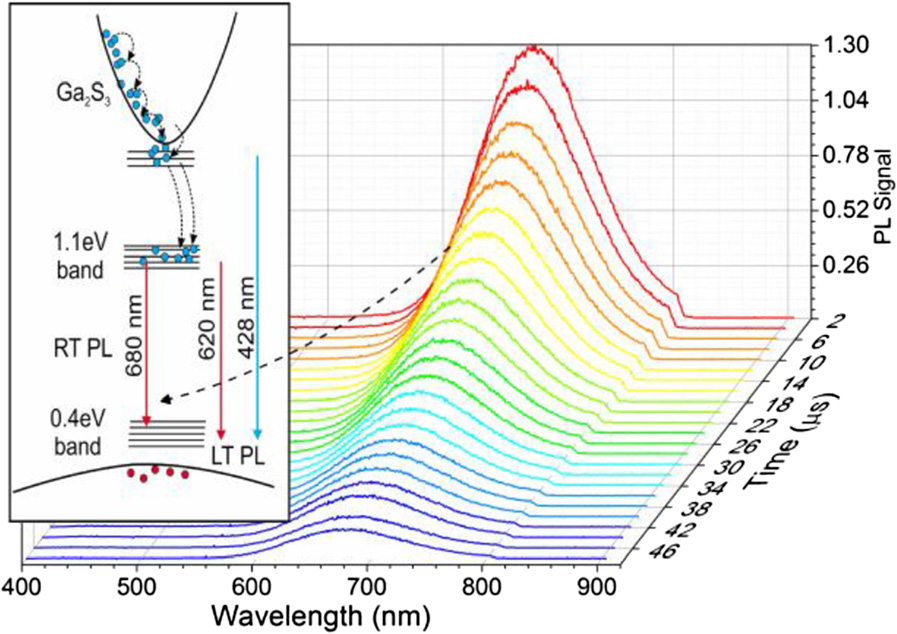
TRPL of the β-Ga_2_S_3_/Ga_2_O_3_ NWs on Si(001) obtained under H_2_S at 500 °C using *λ*_*E*_ *=* 266 nm. The *lower inset* shows a representation of the energy diagram for RT and LT PL

**Fig. 7:**
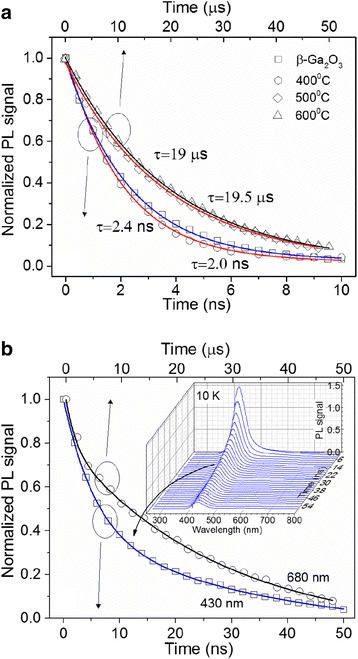
**a** The time evolution of the peaks and fits for all the samples at room temperature. **b** The time evolution for the 430 and 680 nm peaks present at 10 K for the β-Ga_2_O_3_/Ga_2_S_3_ NWs obtained at 500 °C

In order to investigate the carrier dynamics on a longer time scale in the β-Ga_2_O_3_/Ga_2_S_3_ NWs, transient PL measurements were obtained using *λ*_*p*_ = 266 nm and 100-fs pulses. Room temperature (RT), transient, or time-resolved PL (TRPL) of the as-grown β-Ga_2_O_3_ NWs reveal a single exponential decay with a time constant of 2.4 ns. We should also point out that TRPL at 10 K gave an identical decay and a twofold increase in the PL intensity. Similarly, RT TRPL of the β-Ga_2_O_3_/Ga_2_S_3_ NWs exposed to H_2_S at 400 °C consists of a single exponential decay with a time constant of 2.0 ns. On the other hand, TRPL measurements at room temperature of the β-Ga_2_O_3_/Ga_2_S_3_ NWs obtained at 500 °C show a single exponential decay at 680 nm with a much larger time constant of 19 μs (Fig. 6 and Fig. 7a).

The corresponding TRPL at 10 K reveals a new peak at 430 nm following a double exponential decay with time constants 6 ns (62 %) and 42 ns (38 %) (Fig. 7b). The red PL emission can be expressed by a double exponential decay with time constants of 1.8 μs (32 %) and 26.9 μs (68 %) (Fig. 7b). Similar results have also been observed for the β-Ga_2_O_3_/Ga_2_S_3_ NWs at 600 °C. The 26.9-μs time constant is mainly associated with the red emission, and as expected, it is longer at low temperature whereas the short time constant is most likely associated with carriers moving into another state most likely non-radiative.

## Conclusions

In conclusion, we have grown β-Ga_2_O_3_ NWs at 800 °C which were converted to β-Ga_2_O_3_/Ga_2_S_3_ NWs by post growth processing under H_2_S between 400–600 °C for 60 min. We find that the as-grown β-Ga_2_O_3_ NWs exhibit state filling following carrier excitation for probing wavelengths between 340 to 450 nm corresponding to states lying energetically below the conduction band edge. In addition, the β-Ga_2_O_3_ NWs exhibited broad PL with a maximum at 435 nm (2.85 eV) and lifetime of 2.4 ns. Similar results have also been observed in the case of β-Ga_2_O_3_/Ga_2_S_3_ NWs obtained under H_2_S at 400 °C. In contrast, the β-Ga_2_O_3_/Ga_2_S_3_ NWs obtained under H_2_S at 500 to 600 °C exhibit state filling for *λ*_p_ < 450 nm related to the formation of Ga_2_S_3_ and free carrier absorption for *λ*_p_ > 500 nm related secondary excitations from formation mid-gap states in Ga_2_S_3_ located 1.1 eV below the conduction band edge. Furthermore, this is accompanied by a suppression of the broad PL at 435 nm and the emergence of strong emission at ≈ 680 nm (1.8 eV) with a much longer lifetime of 19 μs. This long-life emission is associated with the transition of carriers from the 1.1 eV band to a band located at 0.4 eV above the valence band edge of Ga_2_S_3_. Finally, we like to point out that the room temperature red emission of the β-Ga_2_O_3_/Ga_2_S_3_ NWs may be used in various applications including sensors, non-toxic biomedical imaging, and energy down-conversion in nanowire solar cells.

## Abbreviations

NWs: nanowires
PL: photoluminescence
MO: metal oxide
SEM: scanning electron microscope
*λ*_E_: excitation wavelength
*λ*_p_: probing wavelength
RT: room temperature
LT: low temperature
